# A citation-analysis of economic research institutes

**DOI:** 10.1007/s11192-012-0850-2

**Published:** 2012-10-06

**Authors:** Rolf Ketzler, Klaus F. Zimmermann

**Affiliations:** 1 Bonn University, Bonn, Germany; 2 IZA, P.O. Box 7240, 53072 Bonn, Germany

**Keywords:** Citation analysis, Rankings, Scientometrics, Publication analysis, Economic research institutes

## Abstract

The citation analysis of the research output of the German economic research institutes presented here is based on publications in peer-reviewed journals listed in the Social Science Citation Index for the 2000–2009 period. The novel feature of the paper is that a count data model quantifies the determinants of citation success and simulates their citation potential. Among the determinants of the number of cites the quality of the publication outlet exhibits a strong positive effect. The same effect has the number of the published pages, but journals with size limits also yield more cites. Field journals get less citations in comparison to general journals. Controlling for journal quality, the number of co-authors of a paper has no effect, but it is positive when co-authors are located outside the own institution. We find that the potential citations predicted by our best model lead to different rankings across the institutes than current citations indicating structural change.

## Introduction

In the last decade, German economic research institutes have undergone remarkable changes initiated by the German Scientific Council (Wissenschaftsrat 1998), who requested in 1998 a strong academic foundation of qualified policy advice. Providing high quality research is of great importance and has become one of the dominating endeavours at the institutes in recent years. Since 2000, this request of the German Scientific Council encouraged the institutes to increase the number of peer-reviewed publications. The close connection of academic research and policy advice has emerged to a unique feature of the institutes. In order to establish this strong scientific environment, the institutes strengthened their cooperation with neighbouring universities among others through joint appointments, expanded both national and international research networks, and, at some institutes, established own doctoral programs. One of the key factors behind a successful transformation process was the appointment of new leadership at the institutes.

Public interest in the work of the economic research institutes is generally high due to their high visibility in the media. For this reason, the successful development of the institutes usually notified through rankings was acknowledged beyond the scientific community. Research rankings have become a popular tool for the evaluation of research performance on both individual and institutional levels. Cardoso et al. (2010) have used individual data analysis to compare trends in publication success among labor economists between Europe, the US, and other world regions. Rankings are increasingly used for funding allocation and are gaining increasing influence in tenure appointments. According to Graber et al. (2008), publication records are becoming more relevant for tenure decisions and are likely to further increase in value in the near future. In addition, rankings are a good instrument to improve the national and international visibility of research institutions.

In a recent study Ketzler and Zimmermann (2009) assessed the publication output of the economic research institutes by constructing different rankings for the institutes publication output based on the number of articles published. While research output improved and is about to meet international standards (given a benchmark of one publication per researcher per year at some of the institutes) the reception of the research by the scientific community was not analyzed beyond using simple journal impact factors. Therefore, the innovation of this study is to provide a complete and detailed assessment of the quality of the research output of the institutes on the basis of the citations of the published articles.

Two different approaches have emerged in the literature to evaluate the quality of publications. The first approach relies on the ranks of the journals as an indicator for evaluating the quality of the publication record. This approach is among others based on journal impact factors. A frequently used weighting scheme for scientific journals is provided by Combes and Linnemer (2003). For Germany, Schneider and Ursprung (2008) on behalf of the German Economic Association (Verein fuer Socialpolitik, VfS) classify journals into six categories. Rankings based on this classification appear regularly in the *Handelsblatt*, a leading German business newspaper. The Tinbergen Institute provides a similar classification of academic journals.

The second approach uses citations of a journal article for measuring research quality of the individual article. The number of citations is an indication for academic relevance as well as the international recognition of the research.^1^ This also accounts for the fact that articles published in top journals are often hardly cited, and lower ranked journals often have a more equal share of cites across published articles (Oswald 2007). A greater citation frequency indicates that research results are used by other scientists. This suggests that the contribution extends the scientific frontiers. Given a number of shortcomings of general journal rankings and recognizing that there is an inaccurate relationship between the reputation of a journal and the citation frequency of an article, we follow the second approach.^2^

A number of citation-based studies analyze the research output of university faculties as well as individual researchers, which demonstrates that this approach is becoming more and more the standard practice. An example of a worldwide ranking of economics departments is provided by Coupe (2003). For Germany, Sternberg and Litzenberger (2005) rank faculties of economics and social sciences employing publication and citation levels. An analysis based on citation counts is used by Ursprung and Zimmer (2007) for ranking of German academic economists. Rankings are also produced frequently in other disciplines. Grohmann and Stegmann (2005) construct a ranking for German medical faculties. Another field of research analyses the determinants of the number of citations a paper has received. A recent study of this kind is Hudson (2007). Medoff (2003) addresses the question whether co-authorship among researchers impacts research quality. Laband and Tollison (2000) also examine the impact of collaboration on citation success.^3^

While we use an updated data set of Ketzler and Zimmermann (2009), the very novel feature of this study is to analyze the number of citations econometrically and use it for predictions. The citation analysis is based on publications in peer-reviewed journals listed in the Social Science Citation Index for the 2000–2009 period. We evaluate the impact of the length of the paper and the journal quality and study whether scientific cooperations (co-authorships) affect the number of citations. Given the purpose of our study, we further address the importance of various characteristics of the institutes. As German economic research institutes are now operating in a more competitive environment, we evaluate if the research output differs among the institutes with respect to quality. In addition, we focus on the impact of different research fields on the number of citations as research by institute is, to some extent, interdisciplinary.

In general citation rankings are, in contrast to rankings of the publication output, not directly comparable because an older article typically holds a greater stock of citations than a younger article. This is of great importance since the publication output of the institutes follows different time patterns. The relevance of simple citation counts is therefore limited. A reasonable approach is followed by Grohmann and Stegmann (2005), who present a sequence of citation rankings, each comprising two subsequent years. For constructing the ranking, the authors use observed and expected citation rates, which are based on journal impact factors. We introduce a different method. By eliminating the time dependence of citations our approach allows an evaluation of the entire stock of citations and provides a ranking of the total institutes’ citation success.

## Data and general survey

We evaluate the citations achieved in January 2011 of Social Science Citation Index (SSCI) journal articles published in 2000–2009 by staff members of the German economic research institutes. The analysis focuses on institutes that are members of the Leibniz Association (WGL), which includes the Kiel Institute for the World Economy (IfW), the ifo Institute for Economic Research (ifo), the Halle Institute for Economic Research (IWH), the Rhineland-Westphalian Institute for Economic Research (RWI), the Centre for European Economic Research (ZEW) and the German Institute for Economic Research (DIW Berlin). Our study also takes into account publications of the Hamburg World Economic Archive (HWWA), which was a member of the WGL until 2006. Part of the research is now being continued by the privately financed Hamburg World Economic Institute (HWWI).

The publications used in this study are originally identified using the annual reports of the institutes or, if not available there, from their websites for the period from 2000 to 2009.^4^ The data are compared and, if appropriate, corrected using the ISI Web of Knowledge database. After determining the SSCI output published by the institutes’ scientists, the citations for these articles are recorded using the Journal Citation Report (JCR).^5^ Given the purpose of this study the SSCI index is a natural benchmark for our analysis as the number of citations recorded in the JCR covers only publications in these journals.

In addition, the SSCI index guarantees a high standard of quality in academic journals through strict admission criteria and is characterized by its interdisciplinary nature.^6^ This feature is particularly important since institutions like the research institutes considered here require such a broad scientific basis given the interdisciplinary nature of many different research topics.^7^ Other journal selections cover only the mainstream of economic research. This applies in particular to *EconLit* which is used for rankings in the German scientific community following a recommendation by the German Economics Association (VfS).^8^ However, according to our assessment, using *EconLit* as a journal reference list will lead to distortions in the measurement of the research output in our context since it ignores multidisciplinarity in some of the institutes.

In order to carry out a clearly defined evaluation of the scientific impact of the institutes’ publication output, our study only includes publications by the institutes’ regular employees and its mid-term scholarship holders. Publications by so-called (external) fellows and short-term visiting fellows who list an affiliation to the institutes in their publications are, as a rule, not taken into account. In general, visiting fellows provide research impulses for the scientific activities at the institutes, but their research is usually not conducted in the institutes. In case we are in doubt about the affiliation, we use the CV of the respective individual to identify the position of the researcher.

In a related paper, Ketzler and Zimmermann (2009) have previously examined the publication activities of Germany’s economic research institutes in the SSCI index from 2000 until 2006. We update their analysis for 2007–2009. In 2000, only a few publications in SSCI journals could be counted that account only for 2 % of the total output. Since then, a continuous rise is observed. In 2006, the publication output in SSCI journals was nearly six times higher than in 2000. This trend continues beyond 2006 with an additional 50 % increase in SSCI publications until 2009. In absolute numbers, the publication output in 2009 with 207 SSCI publications is over eight times higher than at the beginning of the period when just 23 articles were published. The increasing publication output across all institutes can also be observed at the level of the individual institutes.

**Fig. 1:**
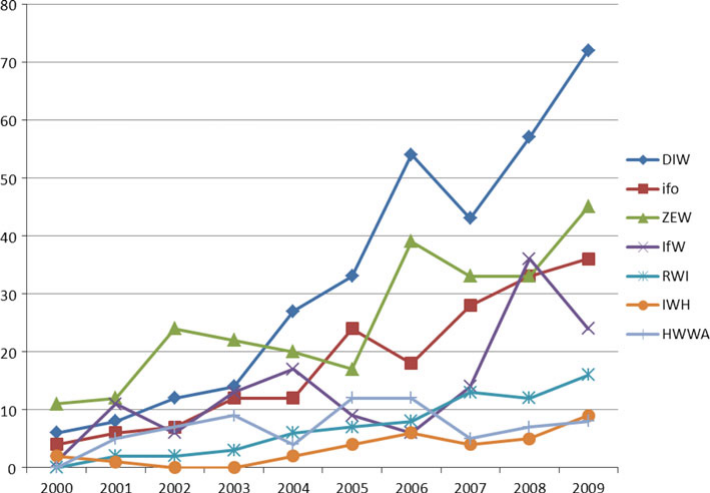
Number of SSCI publications

Figure 1 exhibits the time series of the publications in SSCI journals for the full period 2000–2009 for all institutes. DIW Berlin now dominates the field followed by ZEW, which ranks first from 2000 until 2003. Ketzler and Zimmermann (2009) found that the trends shown appear to be fairly robust when applying different weighting schemes for the publication output, in particular when the data are controlled for impact factors and co-authorship. Some differences in the development can be seen when the publication output is measured in relation to the number of researchers at the institutes, which is an indicator for the productivity.^9^

Table 1 reports descriptive statistics (mean, standard deviation) of the articles used in our analysis. Overall, we cover 1,050 articles in SSCI journals that were cited 4,902 times through January 2011. Hence, the average number of cites per article is 4.7. The first set of variables deal with the allocation of publications between institutes (DIW, HWWA, IfW, ifo, IWH, RWI, ZEW), journal publishers (Blackwell, Elsevier, Oxford, Sage, Springer, Taylor, Other) and across years (2000–2009). The shares of total articles of the institutes across the period are: DIW (30 %), ZEW (23 %), ifo (17 %), IfW (13 %), RWI (7 %), HWWA (7 %) and IWH (3 %). Across time, the share in total output rises steadily from 2 % in 2000 to 20 % in 2009, a rise that marks the amazing success achieved. Elsevier is the most important publisher (28 %) for the research output of the institutes, which results from the large market share in the journal business of this publisher. The next ones are Blackwell (13 %), Springer (12 %), Taylor (8 %), Oxford (3 %) and Sage (2 %), leaving (35 %) for others.

**Table 1:** Published articles

Variable	Mean	SD
Citations	4.669	8.415
Institute (share of articles)		
DIW	0.302	0.459
HWWA	0.067	0.250
IfW	0.126	0.332
ifo	0.172	0.378
IWH	0.031	0.175
RWI	0.068	0.251
ZEW	0.234	0.424
Year of publication (share of articles)		
2000	0.022	0.146
2001	0.041	0.198
2002	0.054	0.227
2003	0.069	0.253
2004	0.083	0.276
2005	0.102	0.303
2006	0.129	0.335
2007	0.130	0.336
2008	0.174	0.380
2009	0.197	0.398
Journal Publisher (share of articles)		
Blackwell	0.129	0.335
Elsevier	0.277	0.448
Oxford	0.029	0.167
Sage	0.018	0.133
Springer	0.120	0.325
Taylor	0.081	0.273
Other	0.347	0.476
Other variables		
Manyauthors	0.725	0.447
Divauthors	0.570	0.495
Economics	0.736	0.441
Fields	0.690	0.463
Pages	18.425	8.568
Impact	0.791	0.659
Limit	0.483	0.500
Number of articles (Nobs)	1,050	
Total cites	4,902	

Published articles, 2000–2009. Number of cites evaluated on January 2011. Pages: average pages published; Impact: SSCI impact factor of journal; other variables are (0,1) dummies

Other variables contained in Table 1 are papers (1) with more than one author (Manyauthors), (2) authors from different institutions (Divauthors), (3) published in economics journals (Economics), (4) published in field journals (sub-areas of economics like labor or energy), (5) with number of pages (Pages), (6) with journal SSCI impact factor (Impact), and (7) published in journals with page limits (Limit). While Pages and Impact are continuos variables, all other variables are (0,1) dummies.

Remarkably, joint production of research is of great importance for the institutes. Almost three quarters (73 %) of all published papers have more than one author (Manyauthors).^10^ 57 % of all articles are joint publications that rely on cooperation with researchers from different institutions (Divauthors). The interdisciplinary nature of the research at the institutes is expressed through the high share of articles published in SSCI-journals that are not listed in the economics sub-field. Most articles (69 %) are published in field journals (Field) reflecting the fact that most researchers in the institutes work in field-specific research departments. Only 74 % of all articles are published in economics journals according to the SSCI classification (Economics). Whereas the average published article has 18 pages with a standard deviation of 9 pages (Pages), practically half of all of them (48 %) are published in journals with page limits (Limit). The average weighted impact factor for the journals of the published articles is 0.79 (Impact).

The average impact factor is 0.73 for the articles that are published in economics journals, while the total average in our data is 0.79. This reflects the known fact that the field of economics cites less than others in the social sciences like sociology and demography. For 2009, the year with the highest publication output, the average impact factor for articles published in economics journals is 1, indicating also a strong rise in average quality over time.

## A ranking analysis by citations

The aim of this study is to provide a citation-based research evaluation. The pure analysis of the publication output using a variety of indicators does not give a complete picture of the institute’s research performance. The quality of a published article is measured only unprecisely by the impact factor of the respective journals (Oswald 2007). For assessing the specific quality of an article, we use its citation frequency. We consider the number of citations of an individual article as a signal for the article’s contribution to new scientific insights. We measure the realized stock of citations in January 2011 for all papers in our database. On average each article published in the period from 2000 to 2009 was cited 4.7 times. We do not adjust the number of citations for the age of the publication.^11^ Therefore, the results presented in this section exhibit an age-related bias, because publications acquire more citations over time.

Table 2 summarizes the total citation counts for the period investigated. For each institute the table contains measures for absolute and relative citations and market share for absolute numbers. According to the first row, ZEW holds the leading position with 1,511 citations, followed by DIW Berlin (1,189 citations), ifo (753 citations), IfW (593 citations), HWWA (384 citations), RWI (382 citations) and IWH (90 citations). The ranking remains unchanged if co-authorship is taken into account (third row): Here we weight the publications using the share of authors from the own institution. The ranking is also unchanged if we use the market share among the cites (fourth row), which suggests that ZEW has 31 % of the cites, DIW 24 %, ifo 15 %, IfW 12 %, RWI and HWWA 7.8 % each, and IWH 1.8 %.

**Table 2:** Citation analysis (as of January 2011)

	DIW	ifo	ZEW	IfW	RWI	IWH	HWWA
Number of citations							
Citations 2000–2009^a^	1,189	753	1,511	593	382	90	384
Citations per article^b^	3.75	4.16	6.14	4.49	5.38	2.73	5.49
Citations 2000–2009 (weighted by institutions)^c^	670.5	475.6	1114.4	103.8	213.8	59.3	247.3
Market share (%)							
Citations 2000–2009^a^	24.3	15.4	30.8	12.1	7.8	1.8	7.8

Articles in journals form 2000–2009. The market share is calculated as the institute's publication output divided by the total output of all institutes in SSCI journals

^a^Number of citations

^b^Number of citations per article

^c^Number of citations weighted by institutions (excluding co-authors from different institutions)

Of further interest are the number of citations per article. We obtain the second row in the Table 2 by dividing the number of citations in January 2011 by the number of published related articles in 2000–2009. Again ZEW ranks first with 6.1 cites per article followed by HWWA (5.5 cites), RWI (5.4 cites), IfW (4.5 cites), ifo (4.4 cites), DIW (3.8 cites) and IWH (2.7 cites).

However, these results are biased since the analysis ignores the very different age distribution of the articles between institutes. We present below in “A ranking analysis using predicted citations” an approach that allows us to compare the number of citations for articles even when they are published in different years.

The distribution of all citations achieved in January 2011 for all papers published in 2000–2009 by all institutes classified by the journal’s year of publication is recorded in Table 3. All articles have had at least 2 years time to be cited, but may or may not get different attention over time. In 2000, all institutes published 23 articles in total, which have been cited 353 times by January 2011. These citations are 7.2 % of all citations received for papers published in the 2000–2009 period. The average cites of papers from that year is 15.4 with a standard deviation of 21. The absolute number of citations is highest for articles published in 2003 (713 citations) followed by a second peak of published articles in 2005 (706 citations). Since then, the number of citations continuously decreases to 193 citations in 2009, which is the lowest for all 10 years. The mean number of citations per article for papers published in 2000 amounts to 15 and is highest for the whole period of our study.^12^ Table 3 also shows a falling number of average citations per article in time (with the exception of 2002 and 2003) to 0.93 in 2009. The average annual citation numbers are well above the results of Sternberg and Litzenberger (2005) for university faculties, who report average citations in the range of 5.6–0.19 for the period 1993–2002.

**Table 3:** Citations in January 2011 according to year of publication

Year	Numbers^a^	Citations^b^	Share^c^	Mean^d^	SD
2000	23	353	0.072	15.348	21.345
2001	43	257	0.052	5.977	8.768
2002	57	466	0.095	8.175	11.149
2003	72	713	0.145	9.903	12.395
2004	87	576	0.118	6.621	9.343
2005	107	706	0.144	6.598	12.267
2006	135	653	0.133	4.801	5.152
2007	136	509	0.104	3.743	5.217
2008	183	476	0.097	2.601	3.317
2009	207	193	0.039	0.932	1.674
	1,050	4,902	1.000		

The large mean in 2000 is based on the very large number of cites received only by the three articles of Sinn (2000) with 41 cites, Gang and Zimmermann (2000) with 43 cites and Puhani (2000) with 84 cites for ifo, DIW and ZEW. Sinn and Zimmermann have been Presidents of their institutes at that time, and Puhani’s article while written in Mannheim at the ZEW was related to his thesis supervised by Zimmermann (and completed at the University of Munich, where Sinn and Zimmermann were both professors). The research of Gang and Zimmermann (2000) was also done during the tenure of Zimmermann as a Professor in Munich. So all three articles are related to the University of Munich and to Mannheim, where Sinn and Zimmermann were both faculty members before their time in Munich

*SD* standard deviation

^a^Number of articles published in the respective year

^b^Number of cites until January 2011 received by articles published in that year

^c^Share of cites achieved until January 2011 received by articles published in that year

^d^Average number of cites achieved until January 2011 per article published in that year

## Econometric analysis

In order to identify the determinants of citation success, we specify an empirical model for the analysis. Since the dependent variable of interest is the number of cites per published article, a count data regression model seems to be an appropriate choice. The baseline model of count data is a Poisson regression, which imposes equality of the variance of the data to its mean, an assumption that often does not hold in real data. Typically, the variance in real applications is larger than the mean, which is called overdispersion, and leaves the Poisson model with too low standard errors. The negative binomial model is the most popular approach to deal with overdispersion. Due to the specific nature of the citation data, which is characterized still by a large number of papers with zero citations and a high degree of overdispersion we use a negative binomial regression model with robust standard errors.^13^

The dependent variable is the number of citations received in January 2011 by SSCI articles published by members of the institutes between 2000 and 2009. Our regression equation includes the number of pages for each article (Pages) as the length of an article is widely regarded as an indicator for scientific substance.^14^ The average quality of the journal in which the paper is published is measured by the SSCI impact factor (Impact), which may suggest additional evidence for the individual quality of the article, but may also predict a better recognition by the scientific community. For both variables we add the squared terms (Pages squared, Impact squared). We are also interested, if a limit set by the journal regarding the maximum number of pages or alternatively words increases the quality of journal articles (Limit).

The model also includes a number of dummy variables to capture additional characteristics of the respective scientific work. First, we relate the number of citations to different journal selection criteria that are used for the evaluation of research performance. Thereby, a number of issues are addressed. We analyze if articles published in general interest journals are significantly more cited than papers in so called field journals (Field). Given the interdisciplinary nature of the research at the institutes and the various disciplines covered by the SSCI, we classify the research output whether it is published in an economics journal (Economics) according to the SSCI index, or if the journal belongs to another sub-index. Second, we analyze if collaboration with other researchers, which is believed to produce higher research quality and hence impacts the quality of the published research. A dummy variable for co-authorship is included which indicates that a paper has more than one author (Manyauthors). As our study focuses on the research quality of research institutes, we investigate if co-authorship with researchers from other institutions like universities enhances the quality of an article (Divauthors).

The overall scientific output of the institutes in peer-reviewed journals has increased remarkably since 2000. While some institutes made a greater contribution to this development, as indicated in Fig. 1, we are also interested if there are differences in the quality of the research within the group of analyzed institutions. To allow for a possibility of a publisher bias, we include dummy variables for the main journal publishers (Elsevier, Oxford, Sage, Springer, Taylor) and others using Blackwell as a reference. We also include dummies for the institutes (HWWA, IfW, ifo, IWH, RWI, ZEW), using DIW as a reference. Finally, we control for the year of publication (Year).

Using the detailed data for all articles in our sample, Table 4 reports the results of our regression model. Most of the year effects are strong and statistically significant, and while all negative with rising size in absolute terms indicating a strong age effect.^15^ We will deal with this issue further in “A ranking analysis using predicted citations”. Our negative binomial count data model provides a strong alpha coefficient capturing the expected overdispersion in the data. The danger of overdispersion, too small standard errors of the estimates of the coefficients, is therefore somewhat covered, but we also provide robust standard errors to deal with the issue.

**Table 4:** Negative binomial model for citations of SSCI publications of German Economic Research Institutes, 2000–2009

	Coef.	Robust Std. Err.	*z*	*P* > *z*	[95 % CI]	
HWWA	−0.081	0.156	−0.520	0.603	−0.388	0.225
IfW	0.162	0.122	1.330	0.185	−0.078	0.402
ifo	−0.124	0.115	−1.080	0.280	−0.350	0.102
IWH	0.162	0.334	0.490	0.626	−0.491	0.816
RWI	0.126	0.153	0.820	0.410	−0.173	0.425
ZEW	0.199	0.098	2.040	0.041	0.008	0.390
Pages	0.040	0.012	3.460	0.001	0.017	0.063
Pages squared	−0.0003	0.0002	−1.670	0.095	−0.001	0.000
Impact	1.417	0.140	10.100	0.000	1.142	1.692
Impact squared	−0.181	0.031	−5.800	0.000	−0.242	−0.120
Limit	0.315	0.082	3.820	0.000	0.153	0.476
Elsevier	0.200	0.131	1.530	0.126	−0.056	0.457
Others	−0.164	0.125	−1.310	0.190	−0.408	0.081
Oxford	−0.339	0.247	−1.370	0.169	−0.823	0.145
Sage	−0.917	0.352	−2.600	0.009	−1.607	−0.227
Springer	0.550	0.157	3.500	0.000	0.242	0.858
Taylor	0.045	0.174	0.260	0.798	−0.297	0.386
Manyauthors	0.112	0.114	0.990	0.324	−0.111	0.335
Divauthors	0.193	0.100	1.930	0.054	−0.003	0.389
Economics	0.042	0.097	0.440	0.663	−0.148	0.233
Field	−0.210	0.101	−2.070	0.039	−0.408	−0.011
y2001	−0.695	0.360	−1.930	0.053	−1.400	0.010
y2002	−0.518	0.330	−1.570	0.116	−1.164	0.128
y2003	−0.391	0.331	−1.180	0.237	−1.039	0.257
y2004	−0.947	0.321	−2.950	0.003	−1.576	−0.317
y2005	−1.068	0.317	−3.370	0.001	−1.689	−0.447
y2006	−1.377	0.313	−4.400	0.000	−1.990	−0.763
y2007	−1.627	0.314	−5.180	0.000	−2.243	−1.012
y2008	−2.316	0.311	−7.440	0.000	−2.926	−1.706
y2009	−3.353	0.318	−10.560	0.000	−3.976	−2.730
Constant	0.928	0.409	2.270	0.023	0.126	1.731
α	0.852	0.055			0.750	0.968
Nobs	1,050					

Likelihood-ratio test of α = 0: $\bar{\chi }^{2}$(01) = 2,579.03 *P* ≥ $\bar{\chi }^{2}$ = 0.000. Reference categories are DIW, Blackwell and 2000

*Nobs* number of published articles

The length of a paper, as well as the journal quality measured by the impact factor, has a significantly positive impact on the number of citations. As is indicated by the signs of the coefficients of the squared terms of the variables the positive effect of the impact factor is decreasing with paper size or impact factor. These results of the regressions are in line with the existing literature. We also find that a journal setting a page or word limit for an article experiences a positive impact on the quality of a published article (Limit).

Our analysis provides no evidence that the publication of an article in an economics journal (according to the SSCI classification) exhibits a significant influence on the number of citations (Economics). In spite of the large share of non-economics research at the institutes, our finding does not support the general observation that different disciplines exhibit different citation standards. However, from the perspective of the economic research institutes, one should note that research results published in non-economics journals are equally acknowledged beyond the economics profession as is economics research in its field. This indicates that interdisciplinarity is well established at the institutes. It does not “hurt” to publish in the journals of other disciplines. But one may also argue that there is no “advantage” to publish outside the own discipline, either because one receives lower attention among the others readers of those journals or the work submitted is of lower quality. Finally, the evaluation has to take into account that the journal impact factor included in the regression already covers some differences across the disciplines.

We provide evidence that publications in field journals (Field) are significantly less cited than articles in journals that cover all branches of economics, although the journal impact factor included in the regression should have taken up already some effects. For the applied policy-oriented research of the institutes, field journals are the natural focus as well as a key element of the publication strategies. This strategy, however, leads to fewer citations than publications in general interest journals, which are to some extent associated with top journals. For the institutes it can be regarded as a success that research results—as far as it is possible—are published in general interest journals, which are very well acknowledged in the academic field, expressed through higher citation numbers. Publications in general interest journals are, in addition to the pure number of publications, an important tool for increasing the visibility of the institutes in the scientific community and should have an important part of the institute’s publication strategy.

Our results suggest that there is a difference in the quality of the research of the institutes whereas DIW Berlin serves as the reference category left out in the estimation. Publications from ZEW researchers are significantly more cited than articles published by researchers from DIW Berlin and all the other institutes controlled for all the other factors included in the regression. This citation success of ZEW researchers is shown through the high number of citations per article in Table 2 as well as the development of the research performance in Fig. 1. Given the time dependence of citations this substantial research output in the first years lead to the high number of citations. ZEW’s leading position in absolute and relative citation numbers results in a qualitative advantage. In contrast, the publication output of DIW Berlin and ifo was initially at a very low level replaced by high growth rates in the middle of the sample period. This result suggests that an increasing publication output is not immediately followed by rising quality. Improving the research impact is in general a medium-term task, which requires an established research culture. Setting up a competitive, high quality academic environment depends on the implementation of a number of different measures that are not effective in the short term. Being by far the youngest of the seven research institutes, ZEW was set up from the beginning to combine high academic quality with strong policy advice activities. This might explain why unmeasured academic credibility has been higher for ZEW research than for other institutes.

Interestingly, there is evidence of a publisher bias in this study. The estimated regression in Table 4 contains publishers by dummy variables and treats Blackwell as the reference publisher. All in all, publications in Springer—journals are significantly more cited than publications elsewhere, and publishing in Sage journals lead to lower number of cites. Following this observation, researchers from the institutes should have an incentive to choose Springer journals for their publications and avoid Sage journals.

Collaboration of researchers through formal co-authorship is supposed to increase research quality compared to single author research. The empirical evidence is, however, mixed. Our findings are in accordance with Medoff (2003) who rejects the hypothesis that co-authored articles receive more citations than sole authored papers (Manyauthors). While unlike theoretical research, both empirically oriented research and interdisciplinary research are characterized by higher rates of co-authorship, this pattern has no impact on the quality of the scientific output. According to our analysis, co-authorship in general does not increase the research quality but may, for example, be reflected in a productive increase of research, which is itself not captured by our data sample. It may, however, be reflected to some extent in the rising number of publications. Note also that we already control for quality factors like pages, journal impact factor and page limits. To deal with such factors by publishing in high-impact journals and be able to produce quality through size under constraints, may well be a matter of the choice of the right co-authors.

On the other hand, if we focus on collaborations among researchers from the institutes with colleagues from different institutions, co-authorship is statistically significant (Divauthors). From the perspective of the institutes, research quality increases through collaboration with external scholars. This result may reflect the general trend of the globalisation of research involving a strengthening of network activities that leads to an increased number of cooperations. Modern communication technologies facilitate a greater exchange of ideas that leads to improved research quality. However, we also note that the empirical evidence for cooperation with external researchers must be considered in light of the rising research performance at the institutes, as indicated in Fig. 1. At the beginning of the surveyed period, scientific research was at a very low level, which was in part a consequence of the insufficient cooperation between the institutes and the universities. Since then, the institutes have expanded their research activities noticeably.

This development was supported by intensifying the cooperation with universities that enabled the institutes to catch up state-of-the-art scientific methods that are publishable in peer-reviewed journals. The increasing number of joint appointments for example, expresses the increasing cooperation. Establishing networks with external researchers represent a key element in the strategy of the institutes. We also note that the analysis by Medoff (2003) as well as Hudson (2007) is concentrated on a small number of top economics journals, whereas our sample data covers a variety of SSCI journals. This difference in the underlying structure of the papers leaves room for improvement in the quality of the research at the institutes. Therefore, the robustness of the discussed impact on the research quality needs to be further evaluated.

## A ranking analysis using predicted citations

As expected, the age of an article has a significant positive impact on the number of citations. Given the results of our empirical model in Table 4, papers published since 2004 are largely less cited than those articles published earlier. Age-dependence may come from different factors. Papers have to be recognized first, which allows for a slow response immediate after publication; afterwards articles are most likely to be stronger cited in the first years, before the interest in the profession either declines or a paper becomes a classic that is cited many years after publication. Rankings of individual researchers’ citation success are often corrected for the so-called career age effect as experienced scholars receive more citations than their younger colleagues.^16^ Other indices, which are used in research performance analysis, including the approach developed by Hirsch (2005), also require a correction by career tenure for a meaningful ranking. The numbers represented by these indicators are annual averages based on career duration.

At the level of institutions we focus on the evaluation of the research quality for a fixed time period. However, compared to the individual analysis, the scientific output of an institution is likely to change over time for a number of reasons that do not apply to the individual researcher. Publication output is among others influenced by the fluctuation of researchers or a new strategic focus of the institute. In fact, due to the requirements of the Leibniz Association, research output has increased since 2000, but differs across institutes. While the ZEW dominated the field at the beginning of our surveyed period, since 2004 a number of institutes have caught up with the development and show high publication growth rates including DIW Berlin which leads the field in 2009. The high number of SSCI publications since 2004 shown in Fig. 1 indicates that the publication output might be followed by an increasing number of citations in the next years, which has not yet been realized. Given our sample data, any citation based ranking that simply sums up the number of citations will exhibit a bias. A method for analyzing the citation performance is presented by Grohmann and Stegmann (2005), who use so called “citation windows” each covering two subsequent years. Alternatively, expected citation rates, based on impact factors, could be computed for constructing a citation ranking.

**Fig. 2:**
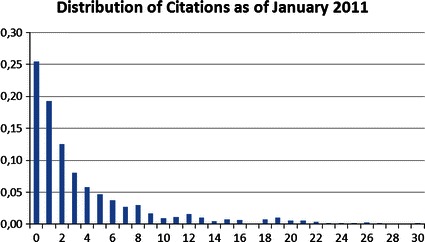
Distribution of citations as of January 2011

**Fig. 3:**
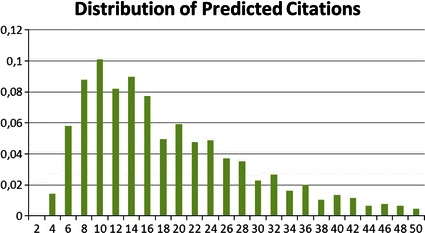
Distribution of predicted citations

In order to establish a ranking for the total citation success of the institutes, we eliminate the time effect by using our regression model in Table 4. Herewith, we predict the number of citations for each article under the assumption that it was published in 2000 by eliminating all year dummies from the model. The prediction output is then the simulated number of citations each paper would have received since the imposed year 2000 by January 2011. Figures 2 and 3 show the distribution of citation counts for the original as well as the predicted data. The observed number of citations follows a right skewed distribution where about 25 % of the papers are not cited (Fig. 2). The skewedness of the distribution is reduced for the predicted values of the number of citations in Fig. 3.^17^

Table 5 exhibits the result of the simulated number of citations per institute for the whole period up to January 2011 assuming that all papers were published in 2000. Overall the predicted number of citations is about 3.5 times higher than the realized citations. For each institute the table contains measures for absolute and relative citations and the market share for the absolute numbers. As is contained in the first row, the simulated number is highest for DIW Berlin with 6,250 citations followed by the ZEW (5,964 citations), ifo (3,575 citations), IfW (2,764 citations), RWI (2,007 citations), HWWA (1,063 citations) and IWH (727 citations). Given the current stock of citations IWH, DIW Berlin and RWI record the highest growth rates with respect to the predicted values. Comparing the simulation with the absolute numbers shows that eliminating the time structure of the citation data involves a clear change in the ranking. While ZEW is the best-placed institute in mere numbers based on the high publication activity through 2003, it falls behind DIW Berlin in the ranking when simulated values are used. This is clear evidence that in particular DIW Berlin can expect papers published since 2004 to be cited strongly.

**Table 5:** Simulated number of citations

	DIW	ifo	ZEW	IfW	RWI	IWH	HWWA
Number of simulated citations							
Citations 2000–2009^a^	6,250	3,575	5,964	2,764	2,007	727	1,063
Citations per article^b^	19.72	19.75	24.24	20.94	28.26	22.03	15.19
Market share (%)							
Citations 2000–2009^a^	28.0	16.0	26.7	12.4	9.0	3.3	4.8

Estimation of simulated number of citations is based on the predicted number of citations from our model in Table 4 under the condition that all papers were published in year 2000. The market share is calculated as the institute’s publication output divided by the total output of all institutes in SSCI journals

^a^Number of citations

^b^Number of citations per article

The ranking is also changed if we use the market share among the predicted cites (third row), which suggests that DIW has 28 % of the cites, ZEW 27 %, ifo 16 %, IfW 12 %, RWI 9 %, HWWA 5 %, and IWH 3 %. The variation in the market shares of predicted compared to actual citation numbers underlines the rising scientific significance of DIW’s publication output, which has not been realized yet. Our model estimates an increasing market share of citations for DIW Berlin by nearly four percentage points, which is the highest increase for all institutes. In contrast, ZEW’s share in the number of all citations drops about the same amount. The publication output of DIW Berlin, which has continuously increased over the sample period, is very well reflected in the ranking of the simulated number of citations representing the total citation success of the institutes from 2000 to 2009.

The relative numbers of citations that result from our simulation provides evidence that there is an overall improvement in the quality of the research at the institutes. On average papers authored by members of the economic research institutes can expect about 21 citations, 12 years after the paper is published. This is an increase by six citations compared to articles that are originally published in 2000 (Table 3). Table 5 also contains an analysis of predicted relative citation indicators. When predicted citations per article are studied (second row), RWI achieves the highest position (28 citations) followed by ZEW (24 citations), IWH (22 citations), IfW (21 citations), ifo (19.8 citations), DIW (19.7 citations), and HWWA (15 citations).

The rankings using the relative citation numbers reveal differences in the publication strategy of the institutes. The high number of citations estimated for RWI’s moderate research output is an indication that the publication strategy is primarily focused on quality. Compared to articles published in 2000, which received 15 citations on average RWI nearly doubles the citations according to our model prediction in Table 5. While ZEW’s research output is characterized by high publication numbers and a sound quality, the majority of the institutes in the midrange of the ranking put up to now a higher emphasis on the quantity of the publications. However, as outlined above at most of the institutes research quality will improve as well, albeit predominantly on a smaller scale. Therefore, the high increase in the number of peer-reviewed articles as experienced at DIW Berlin in recent years will have a positive impact on the quality of the research in the medium term. HWWI now instead of HWWA is identified as a “relegated institution” by the model, falling from second place to behind all other institutes. This result is in line with the new private organisation of the institute with a new focus on policy consulting after losing public funding in 2006.

## Summary and conclusions

German research institutes have undergone a regime shift after 1998, when the German Scientific Council (Wissenschaftsrat 1998) had requested a strong academic orientation for qualified policy advice. This has lead to the proposal that each researcher should publish one article in a refereed journal per year to ensure awareness and quality of the professional policy advisor. Each researcher should do both, academic research and policy advice. While very much under attack inside the institutes and among policy makers, this goal has lead to an amazing rise in publication output from those research institutes over the decade studied here (2000–2009).

The novel feature of the paper is to provide methods to judge the quality of the perceived publication output. This is done by measuring the output by cites received and not by quantity of papers published in the SSCI journals or the ranks of the journals according to average cites per journal. The determinants of cites for each article are quantified by employing a count data regression model; in particular we estimate the negative binomial model. The quality of the publication outlet has a very significant, strong and positive effect on the number of cites an article obtains, but the increase is smaller the higher the journal quality is. Individual paper quality, proxied by the number of the published pages, also has a positive effect on cites. But it is also found that articles in journals setting a page or word limit receive a positive impact on the number of citations. Field journals get less cites in comparison to general journals.

Collaborations among researchers, either within the institutes or with actors from outside centers of excellence, play a strategic role in the quality production of the institutes. Our research finds that the number of co-authors has no effect on the number of cites, but it is positive (although not strongly significant) when co-authors are located outside the own institutes for instance in universities. We should note, however, that such results are achieved in a regression analysis that controls for journal quality by average SSCI impact factor.

Further, we have made transparent the existence of employer and publisher bias among the cites of published articles of research staff from the German research institutes: If from the Centre for European Economic Research (ZEW), articles have significantly more cites than if from the German Institute for Economic Research (DIW Berlin) or one of the others. If published in a Springer journal, the articles receive more cites than with Blackwell, and if published with Sage, the articles receive significantly less cites than with Blackwell. The other publishers are not different from Blackwell.

We further find that the calculated potentials from our regression model lead to quite different rankings across the institutes than the current cites are indicating. In the raw data, ZEW holds the leading position with 1,511 citations, followed by DIW Berlin (1,189 citations) and the ifo (753 citations). The simulated number assuming that all output would have been published in the base year 2000 is highest for DIW Berlin with 6,250 citations followed by the ZEW (5,964) and the ifo (3,575) indicating some structural change underway.

Our study has also shown that rankings among the institutes are quite different if cites per articles is the major objective independent of the number of papers published, the structure of the research staff and other objectives like providing policy advice. While a high-quality strategy is valuable if the key object is research and feasible, if the institute is small, this is not a useful strategy in general. The request of research orientation and publication output formulated by the German Scientific Council (Wissenschaftsrat 1998) was driven by the understanding that good policy advice needs academic training, and hence regular publication efforts. There is neither a need for all policy advisors to publish in the best journals of the profession and to get the largest numbers of cites, nor is this possible given the limited space in those outlets.

Scientometric analysis can gain substantially through measurement and quantification. However, the ideal concept is still under debate. While the inclusion of papers published in journals ranked by the SSCI is often used next to the average impact of the respective journals, these measures are also debatable. We have suggested here to use actual and potential cites and have demonstrated its usefulness for a particular example, the ranking of German economics research institutes. However, we think that the suggested approach is also applicable and has promise in other contexts of scientometrics.

## Footnotes

1 The measurement of article quality using citations is discussed in Medoff (2003).

2 A detailed discussion comparing both approaches is given by Schläpfer and Schneider (2010).

3 There is strong evidence in the literature that the number of citations is positively depended on the length of the article and journal quality. The evidence of an impact in regards to the number of authors is however mixed. Medoff (2003) and Hudson (2007) find no empirical evidence that collaboration results in higher quality research. For German economists Ursprung and Zimmer (2007), however, demonstrate a positive impact of co-authorship.

4 Publications in peer-reviewed journals which belong to the Science Citation Index (SCI) or other indices are not taken into account in this study.

5 A small number of papers are not listed in the JCR and are excluded from our dataset.

6 For the same reason the SSCI is also chosen by Sternberg and Litzenberger (2005).

7 Moreover, the institutes have a high share of researchers with a non-economic and interdisciplinary focus, for instance in the field of energy and environmental research as well as socioeconomic issues. Therefore, research results are also published in renowned non-economic journals such as *Energy Policy*, *Electoral Studies*, *International Migration Review*, *Journal of Gerontology*, *Public Opinion Quarterly*, *Sociological Methods and Research * and *Transportation Reviews*.

8 Schneider and Ursprung (2008) report that additional journals will be added to the GEA journal list, if these journals represent important research outlets.

9 For a detailed discussion see Ketzler and Zimmermann (2009). In addition, their analysis provides an approach to measure the inequality of researchers, which contributes to publication activities of the institutes.

10 The high share of joint publications is an incidence for the importance of the empirical-oriented as well as interdisciplinary research at the institutes, where joint research is more common than among theoretical-oriented scholars. Medoff (2003) reports that 49 % of the papers in his data set are co-authored.

11 See however Sect. 5 below for a ranking analysis using predicted citations.

12 See the footnote in Table 3 for a more detailed explanation of this finding.

13 See Winkelmann and Zimmermann (1995) for a survey of count data models. The negative binomial regression model fits the estimated probability distribution better than the Poisson regression model. A likelihood ratio test clearly rejects the hypothesis of a Poisson model at all common confidence levels, whereas a goodness of fit test for the negative binomial regression is not rejected.

14 We do not standardize the page size to account for variations among different publishers, but we control for page size restrictions between journals (Limit).

15 The negative sign of all year coefficients also indicate that the particular performance of some of the papers published in 2000 as discussed in the footnote of Table 3 were not taken up by the regressors.

16 The extent by which the career age influences the ranking of individual researchers is investigated by Ursprung and Zimmer (2007).

17 A comparison between actual and predicted citations provided by Figs. 2 and 3 suggests still substantial differences between them. Additional parameterisation of the overdispersion parameter (as in the generalized negative binomial model) or models allowing the independent variables to separately affect the distribution of citations (e.g. non-linear squares estimation, hurdle models) might be able to address this problem. Cameron and Trivedi (1998) discuss such models.
